# Cardiac magnetic resonance perfusion imaging and the effects of single intravenous cannulation with the Octopus bionector

**DOI:** 10.1186/1532-429X-15-S1-O53

**Published:** 2013-01-30

**Authors:** Heiko E Kindler, Eliana Reyes, Arun J Baksi, Arun Natarajan, Harith Alam, John-Paul Carpenter, Raad Mohiaddin, Peter D Gatehouse, Dudley Pennell

**Affiliations:** 1 CMR Unit, Royal Brompton Hospital, London, UK, London, UK

## Background

CMR perfusion (CMRP) imaging using adenosine traditionally requires bilateral arm cannulation. Patients with multiple comorbidities often have difficult venous access and dual cannulation often proves impossible. We used a standard two-way adapter (Octopus Vygon with no-reflow valve) to administer adenosine at a standard rate of 140 mcg/kg/minute over 3 minutes for maximum coronary vasodilatation following a bolus injection of gadolinium. High flow bolus injection may cause sinus arrest caused by a flush of residual adenosine in the same arm vein. We acquired 50 sequential R-wave triggered image frames to assess first pass myocardial perfusion and assessed the effect of significant sinus pauses on image acquisition.

## Methods

First pass perfusion was performed on a Siemens Avanto1.5T MR scanner (Siemens Medical Solutions, USA) with a standardised acquisition protocol using intra-venous adenosine 140μg/kg/min for 3 minutes. Three sequential short axis slices of 8mm thickness were acquired per cardiac cycle using a hybrid EPI sequence (TR 175 ms, TE 1.03 ms, flip angle 25 degrees and voxel size 2.8 x 2.8 x mm^2^) after administration of a 0.1 mmol/Kg bolus of intravenous Gadolinium (Gadovist). Fifty sequential R-wave triggered frames were acquired to assess first pass perfusion. We assessed the effect of significant sinus pauses on patient safety and quality of image acquisition.

## Results

**Figure 1:**
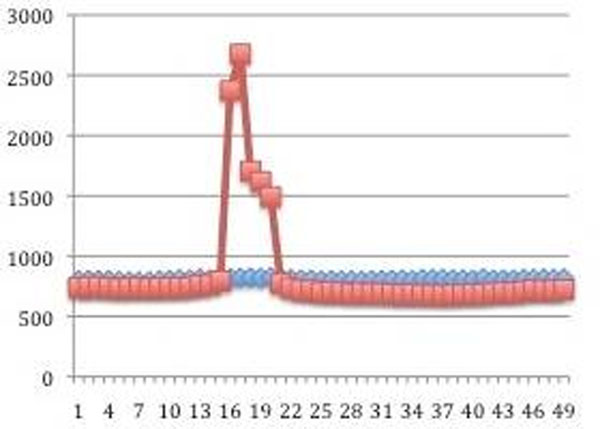
RR interval over 50 image acquisition frames with adenosine (red line) and at rest (blue line) in a typical patient with Octopus Bionector.

**Figure 2:**
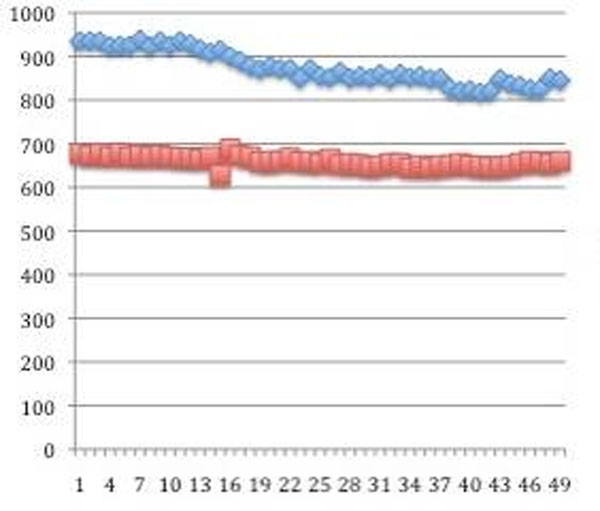
RR interval over 50 image acquisition frames with adenosine (red line) and at rest (blue line) in a typical patient without Octopus Bionector.

One hundred and seven CMRP studies using the Octopus two-adapter were compared with 107 standard method dual vein cannulation for CMR first pass perfusion between May 2012 and August 2012. Twenty seven of 105 patients (25.7% vs 0% in the control group) developed significant episodes of bradycardia. Mean heart rate 69.1(+/-28.1) Range: 139-18 min-1). Image sequences during first pass perfusion were adversely affected due to gaps in R-wave triggered image frame acquisition during, or just before gadolinium arrival because of the effects of adenosine (Figures 1 and 2).

## Conclusions

Crucial image frames are not acquired at the most important time points during first pass perfusion in 26% of patients with the Octopus Bionector. The introduction of a two-way intravenous adapter resulted in an unacceptably high number of patients having sinus pauses/sinus arrest over several seconds, And as image quality is heavily dependent on R wave intervals being regular, this adversely affected image quality. This could have led to underdiagnosis of perfusion defects in the affected patients.

## Funding

N/A.

